# The Intersection of the COVID-19 Pandemic and the 2021 Heat Dome in Canadian Digital News Media: A Content Analysis

**DOI:** 10.3390/ijerph20176674

**Published:** 2023-08-29

**Authors:** Emily J. Tetzlaff, Nicholas Goulet, Melissa Gorman, Gregory R. A. Richardson, Glen P. Kenny

**Affiliations:** 1 Human and Environmental Physiology Research Unit, School of Human Kinetics, Faculty of Health Sciences, University of Ottawa, Ottawa, ON K1N 6N5, Canada; etetz085@uottawa.ca (E.J.T.); gkenny@uottawa.ca (G.P.K.); 2 Heat Division, Climate Change and Innovation Bureau, Healthy Environment and Consumer Safety Branch, Safe Environments Directorate, Health Canada, Ottawa, ON K1A 0K9, Canada; gregory.richardson@hc-sc.gc.ca; 3 Behavioural and Metabolic Research Unit, School of Human Kinetics, Faculty of Health Sciences, University of Ottawa, Ottawa, ON K1N 6N5, Canada; 4 Clinical Epidemiology Program, Ottawa Hospital Research Institute, Ottawa, ON K1H 8L6, Canada

**Keywords:** SARS-CoV-2, extreme heat, climate change, health messaging, public health, intersecting crises

## Abstract

During the 2021 Heat Dome, 619 people in British Columbia died due to the heat. This public health disaster was made worse by the ongoing COVID-19 pandemic. Few studies have explored the intersection of heat with COVID-19, and none in Canada. Considering that climate change is expected to increase the frequency of extreme heat events, it is important to improve our understanding of intersecting public health crises. Thus, this study aimed to explore media-based public health communication in Canada during the COVID-19 pandemic and the 2021 Heat Dome. A qualitative content analysis was conducted on a subset of media articles (*n* = 520) related to the COVID-19 pandemic which were identified through a previous media analysis on the 2021 Heat Dome (*n* = 2909). Many of the articles provided conflicting health messages that may have confused the public about which health protective actions to take. The articles also showed how the COVID-19 pandemic may have exacerbated the health impacts of the 2021 Heat Dome, as pandemic-related public health measures may have deterred people away from protecting themselves from heat. This study, which provides novel insight into the prioritization of public health messaging when an extreme heat event occurs concurrently with a pandemic, supports the need for consistent heat health guidance.

## 1. Introduction

The COVID-19 pandemic has had devastating impacts on the global population and health systems, uncovering vulnerabilities and weaknesses in health services, including health inequities. During the summer of 2021, the COVID-19 pandemic intersected with the 2021 Heat Dome, a prolonged period of abnormally high temperatures that covered a large geographic area of western Canada and the United States, exposing many people to hazardous heat and straining healthcare services [1]. In Canada alone, this extreme heat event (EHE) killed 619 people in British Columbia (B.C.) [2] and an estimated 66 more in Alberta [3] between 24 June and 12 July 2021. In comparison, 18 individuals in British Columbia and 26 individuals in Alberta died of COVID-19 during roughly the same period (24 June 2021, to 21 July 2021) [4,5]. 

The combination of extreme heat and COVID-19 had devastating collateral effects, further compromising public health and severely straining emergency health services. In response to the COVID-19 pandemic, health authorities across Canada implemented various public health measures to limit the transmission of the virus, including physical distancing, masking, lockdowns, vaccinations, and testing centers [6]. However, some of these actions may have inadvertently conflicted with best practices to protect people from extreme heat (e.g., access or capacity restrictions for cooling centers to respect physical distancing mandates) [7]. Additionally, COVID-19 restrictions may have discouraged people from accessing cooling centers or seeking emergency support due to the fear of being exposed to the virus [8]. Therefore, public health interventions for COVID-19 may have disrupted health measures typically implemented in response to extreme heat. 

With climate change projections indicating a rise in the frequency of EHEs [9] and the inevitability of future pandemics globally [10], it is vital to understand the impacts of intersecting public health crises on public health and healthcare systems. In turn, strategies can be developed to improve resilience and reduce health and social support systems’ disruption in future EHEs that occur concurrently with an outbreak or pandemic. Existing investigations into these compounding impacts have primarily explored discrete impacts (e.g., hospitalizations, occupational health [11,12]), but have not examined how the combined effect of systemic social vulnerabilities and pandemic-related factors contribute to extreme heat risk (Table 1). 

Due to the limited sources of data available for exploring the combined impacts of these intersecting public health crises, we sought to address these knowledge gaps by examining references to COVID-19 in digital media articles on the 2021 Heat Dome in Canada published between June 2021 and February 2022. As the media serves as a significant information source to the public [41] and powerful medium for raising public awareness [42], analyzing media articles is one qualitative method that can help capture more details about the compounding health impacts of two or more health crises. Further, exploring news coverage can provide a unique medium for understanding how intersecting crises are presented to the public, as journalists draw on a wide array of sources and perspectives [43,44,45]. Although several analyses of media coverage of individual EHEs have been undertaken globally [43,46,47], few have been conducted within the Canadian context [46], and none in relation to an EHE and COVID-19.

Thus, by harnessing the wealth of media articles circulated to the public during and after the 2021 Heat Dome, this study presents a unique opportunity to evaluate the intersecting impacts of an EHE with an ongoing pandemic in Canada. Findings from this study will assist in providing valuable guidance to public health authorities related to risk prioritization of public health messaging, health protection planning, and identifying vulnerabilities in the public health response to dual crises. Further, the findings have applications beyond the intersection strictly of COVID-19 and heat as they provide a greater theoretical understanding of the combined impacts of two health crises.

## 2. Materials and Methods

This study is part of a larger research project that examines the portrayal and communication of health risks and impacts associated with the 2021 Heat Dome in Canada within digital news articles [48]. A systematic review was conducted for a thorough analysis of digital media content, which included various types of materials such as newspaper articles, blogs, newsletters, community bulletins, municipal meeting minutes, public health unit posts, radio broadcasts, and television transcripts. The search strategy was developed in consultation with a research librarian, and the final search underwent review by a second librarian before database translation (see Supplementary Material for more details).

The search strategy included eight academic databases (Medline, Embase, C.A.B. Abstracts/Global Health, Agricola, FSTA, EconLit, PsycINFO, and Scopus) and five subscription news databases (ProQuest Canadian Major Dailies, Business Source Elite, NewsDesk, Factiva, and Eureka). The scope of the search was limited to articles in English and French published within Canada between 1 June 2021 and 26 February 2022. The objectives of the search strategy were to (i) minimize reliance on prestige press (i.e., The Globe and Mail, Toronto Star, National Post) and limit outlet bias [49] and (ii) capture all news articles published following the forecasted extreme heat alert, during the heat event, and several months after its conclusion, which included subsequent weather events that were made more likely due to the 2021 Heat Dome (e.g., wildfires) [50]. Content from social media platforms like Twitter and Facebook, as well as materials without transcription, such as audio and video-only content, were excluded.

In addition to the database searches, the search strategy included a list of targeted websites belonging to public and non-profit organizations for each province and territory in Canada, including national sites. Given the geographic impact of the 2021 Heat Dome, detailed web searching in the western provinces (i.e., British Columbia, Alberta, Saskatchewan, and Manitoba) was prioritized, focusing on agencies related to health, environment, agriculture, infrastructure, housing, labor, safety, hydro, school boards, municipalities, and Indigenous communities. For the remaining provinces and territories (*n* = 9), the search was simplified to cover health, agriculture, housing, and labor. For each targeted website (*n* = 997), the search terms “heat” and “2021” were entered into the search function. When a search function was not available, the authors (E.J.T. and N.G.) manually searched the website by targeting the homepage, news tabs, newsletters, and publication/resource tabs. Additionally, Advanced Google searches were performed for each province and territory to ensure coverage of open-access online news sources, using the following search string: (“location” AND “heat wave” OR “heat dome” OR “extreme heat” AND “2021”). The Google searches were continued until the following notice was reached: *“in order to show you the most relevant results, we have omitted some entries very similar to the X already displayed.”*

In the larger research project, the authors created a codebook of concepts, positive indicators of a given code, and examples of the dataset. An initial round of screening was then performed to identify relevant articles captured by the complete search strategy (*n* = 152,597). All relevant articles (*n* = 5357) were uploaded to Zotero (Release 6.0, Corporation for Digital Scholarship), a reference manager software, and NVivo (Release 1.6.2, QSR International), a qualitative data analysis software. A trial coding of 500 randomly selected articles (~10% of relevant articles) was completed independently by two authors (E.J.T. and N.G.). A coding comparison query was then performed and revealed that the authors achieved a high percentage of agreement and a kappa coefficient of 0.64 for this sub-analysis, indicating a “good” strength of agreement [51]. The remaining articles (*n* = 4857) were divided evenly and reviewed and coded by the authors (E.J.T. and N.G.). Due to the size of the dataset, full-text review and deduplication co-occurred with coding. All articles included in the larger project’s analysis (*n* = 2909) were then reviewed to identify references to extreme heat and COVID-19 as information-rich cases for this secondary analysis. Terms indicative of the COVID-19 pandemic were determined based on a pre-set list of Medical Subject Headings controlled vocabulary thesaurus terms, including SARS-CoV-2, COVID-19, COVID, and pandemic. The resulting dataset included 520 articles published in Canada between June 2021 and February 2022 (18% of the larger dataset). After coding was complete, the characteristics of the included documents and extracted data (coded findings) were analyzed using a series of NVivo query functions (e.g., date of publication and word frequency). Next, a qualitative content analysis method was used to describe the meaning of the data. The authors then met to discuss the data to reach an agreement on the broader themes and concepts.

## 3. Results

Five main themes were identified within the articles that mentioned both COVID-19 (COVID-19: *n* = 1709; pandemic: *n* = 596; SARS-CoV-2: *n* = 7) and the Heat Dome (Supplementary Table S1). Quotations from the analyzed news articles are used throughout to provide evidence and supplement the themes and concepts identified. Table S1 in the Supplementary Materials provides additional details for each theme, including concepts and their related counts, positive indicators (keywords) related to each concept, as well as additional quotes from the media.

### 3.1. Communicating the Burden of Multiple Intersecting Public Health Crises

Many articles conveyed the burden of being faced with multiple intersecting health crises. For example, one article noted *“first COVID and now this”* [52], and another described the series of cascading natural hazards *“from the global pandemic to opioid crisis to heat dome to atmospheric rivers”* [53]. The media frequently mentioned the various health crises in the context of the 2021 provincial election in British Columbia, emphasizing their influence on voters and election outcomes. In other cases, these references were used to communicate opinions on emergency preparedness in general. For example, *“we are still very much in the throes of dealing with the last crisis, even as the next crisis is upon us—the fallout of climate change. And like the COVID-19 pandemic, we are only partially prepared”* [54]. In contrast, other articles emphasized the demand on the health system, infrastructure, and specific individuals/positions affected by the intersecting crises, as illustrated by the following quotes: *“officials are balancing COVID restrictions with the need for people to stay cool”* [55] and *“the City of Vancouver has implemented additional measures to protect residents facing compounded challenges of COVID-19 and the heat”* [56].

The media articles also communicated the burden of the two crises on health workers and the health system. The texts frequently referred to workers being *“burdened by the crushing COVID-19 pandemic and record-breaking heat wave”* [57] as *“many health professionals were already working seven days a week on COVID-19… The heat wave on top of this [was] almost the straw that could break the camel’s back”* [58]. A few articles reported that staff were shifted from non-emergency care to emergency services to accommodate the demand on the health system. For example: *“nurses are being moved into the hospital’s emergency department to help people deal with the current heat wave, the heavy smoke from wildfires and COVID”* [59]. Concerns were raised about the prolonged wait time for emergency care. For example, one health authority in British Columbia asked that *“people who don’t need emergency care to visit walk-in clinics and primary care centres instead of emergency rooms, as hospitals deal with a spike in patient numbers”* [60]. Emergency medical services across British Columbia reported being extremely strained by three-fold spikes in call volumes during the EHE. On a day when *“Vancouver Fire attended 365 calls, including cardiac emergencies, heat emergencies and overdoses”* [61], some firefighters spent up to 11 hours with a patient waiting for an ambulance.

The mental health impacts on the public from multiple intersecting public health crises were frequently reported (*n* = 47) by the media. For example, one article noted that Canadians had *“been dealing with a lot of grief lately, from the pandemic and opioid health crises…to a deadly heat wave”* [62]. The president of the Paramedic Association of Canada added that *“extreme weather events, like last summer’s deadly heat wave in BC, have highlighted the need for more staff and better mental health supports for first responders”* [63]. In addition to the physically demanding working conditions faced during extreme heat, one paramedic described how mentally taxing the job can become: *“You’re wearing a respirator, you’re wearing a face shield, of course, you’re wearing plastic gloves, and a plastic gown. Even in regular weather, that’s very taxing, but imagine during the heat dome what it would have been like…That sort of plays into your mind, too, because people are upset—‘Come on, hurry up, help’—but you’ve got to protect yourself, too”* [64].

Many articles also discussed the mental health toll in the agricultural industry. For example, one farmer said, *“the stress of this year is piled on top of the stress from the pandemic”* and further commented on being *“worried about the mental health of producers [as] this just adds to the stress because there’s absolutely nothing you can do. You’re watching your crop burn away”* [65]. In the restaurant industry, one business owner closed his doors for a week to give himself and his employees a mental health break: *“it’s not just COVID-19 that’s contributed to unprecedented stress levels for himself and his staff, but also…this summer’s deadly heatwave and yet another harrowing wildfire season”* [66].

### 3.2. Prioritizing Crises and Conflicting Public Health Messaging

The media articles showed conflicting public health messaging and differences in which crises were more prominently covered. While public health authorities were recommending how the public could access cool indoor spaces, a series of articles also suggested that people gather outdoors, despite the extreme heat, terming it *“much safer than gathering indoors”* [55]. In contrast, other articles communicated the opposite, such as: *“we would prefer that people avoid the exposure to extreme heat outdoors…We also realize that after a year-and-a-half of COVID restrictions, if people can take advantage of the weather and gather, they will do it. But it is super important to pay attention when it comes to smoke or heat”* [67].

In the media, public health messaging often prioritized heat protection over COVID-19, suspending existing infectious disease protocols such as masking and physical distancing. For example, one health agency *“clarified that COVID-related occupancy restrictions, physical distancing, and wearing of masks at cooling centres is not required”* [68]. Similarly, another public health agency advised *“that risks from extreme heat exceed risks from COVID-19”* [69] emphasizing that *“COVID-19 protocols take [a] back seat during a heat wave”* [70]. Some articles also quantified this prioritization with headlines like *“global heating surpassed COVID-19 as the existential crisis at the forefront of our thoughts”* [71] and through reporting the difference in deaths between COVID-19 and the EHE. For example, *“In the week before Canada Day, over 700 people in British Columbia died in Western Canada’s record-breaking heat wave—triple the number that would normally occur. In the same period, 10 people in the province died from COVID-19”* [72]. Another comparison emerged when *“the Vancouver School board decided to close all schools…something not even COVID-19 could convince them to do this year”* [71].

Numerous articles cited how prioritizing heat over COVID-19 translated practically into real-time revisions of public health measures to accommodate the difficult circumstances. For example, some reports stated that *“while the Extreme Heat Alert is in place, cooling centres will be open, and no one should be denied access to these centres because of concerns about crowding or physical distancing”* [73]. Similarly, a few articles cited that *“no one should be denied entry to a cooling centre for not wearing a mask”* [74] or that *“if people [were] wearing a mask and have difficulty breathing, they should remove the mask, whether they are indoors or outside, as wearing a mask may impact thermal regulation during heat events”* [75]. These changes to COVID-19 protocols also happened in healthcare settings. For example, *“with respect to the removal of masks within healthcare settings, for the duration of the heat wave, we temporarily recommend allowing patients, clients or visitors to remove masks if they feel it is causing difficulty breathing due to the heat”* [76]. Lastly, some municipalities responded to the EHE by reactivating water fountains that were previously closed due to COVID-19 protocols.

Some media articles raised the *“inadequacy”* of British Columbia’s response to the heat dome in comparison to the province’s COVID-19 response [77]. For example, one opinion article conveyed that *“the B.C. government was so caught up in celebrating the latest phase of the COVID-19 restart last week that it was caught off-guard by the record-setting heat wave and the early start of the wildfire season”* [78]. Of the 11 reports expressing similar sentiments, 10 of them specifically referred to the Premier of British Columbia’s response, noting he was *“a bit giddy at the prospect of saying goodbye to the state of emergency and stepping into the third step of [B.C.’s] reopening plan”* [79]. Articles criticized the Premier’s comments that the government *“didn’t think of it as catastrophic hot weather. We thought of it as hot weather”* and his statements that fatalities are *“part of life”* and that emergency responses involve an *“element of personal responsibility”* [80]. One individual, who lost her grandmother due to the extreme heat, expressed her disappointment in the provincial response to the Heat Dome: *“After 18 months of clearly communicating the risks around the global COVID-19 pandemic, Gaba feels her government failed her Nana and the others who died when it came to doing the same about the heat”* [81].

### 3.3. COVID-19 Exacerbated the Health Impacts of Extreme Heat

Media articles identified that some pandemic-related measures exacerbated heat health impacts during the Heat Dome. For example, *“due to COVID-19 protocols, parents at some schools [had to] wait outdoors temporarily, until going inside to see their child cross the stage [for graduation]. Some ceremonies are expected to last through the afternoon when Burnaby’s temperatures are predicted to be 42 °C and feel like 48 °C with humidity”* [82]. Therefore, by abiding by the COVID-19 restrictions, many individuals may have been exposed to high-risk heat conditions. There were also circumstances where the lack of COVID-19 public health programming in some areas influenced the implementation of heat health mitigation measures. Articles warned of crowding at locations that were not imposing capacity or physical distancing requirements, such as specific cooling centers and air-conditioned public spaces (e.g., libraries, malls, recreation centers). For example, one report cited a City of Regina staff warning *“that these places are expected to be busy, and for those worried about COVID-19 to assess the spaces for themselves. If there is a spray pad in your community that you most often frequent, perhaps plan a backup location if you can. If you go there, it’s quite crowded, and you might not be as comfortable accessing it. Maybe try the second or third one”* [83].

Many articles discussed the requirement for COVID-19 public health measures to be respected during heat mitigation programs and recommended activities. These included: physical distancing (*n* = 80), masking (*n* = 71), ventilation and fan use requirements (*n* = 17), symptom screening at cooling centers and other public spaces (*n* = 10), and enhanced sanitization/cleaning requirements (*n* = 8). The media often portrayed the requirement to abide by these measures as a barrier to accessing various facilities and services (e.g., cooling centers, splash pads, and air-conditioned malls). For example, cooling centers were significantly restricted *“in accordance with current public health orders”* [84], with many offering only 13–15% capacity for people trying to escape the heat (e.g., *“downtown centre’s capacity [was restricted] to only about 45 people, rather than its normal 300-to-350-person capacity”* [85]).

Heat health mitigation measures were reported to be impacted by measures aimed at preventing the transmission of COVID-19. This included the impacts of public health orders on the use of indoor public spaces. For example, one community service provider stated that *“the COVID-19 pandemic has added an extra obstacle because capacity restrictions in many buildings mean there are fewer indoor places to go to cool down”* [86] including libraries and shopping centers. Some outdoor cooling spaces were also subject to COVID-19 restrictions. For example, *“spray parks and splash pads will open but will be subject to capacity restrictions due to COVID-19 restrictions until July 1st”* [87]. Considerations related to the COVID-19 pandemic were also embedded in messages about wellness check procedures for heat exposure monitoring: *“If someone experiences these symptoms, move them immediately to cooler conditions, and have them rest and drink a cool beverage. Wear a mask and make sure you wash your hands before and after helping a loved one you do not live with. Make sure someone from their household can stay with them, and if they do not immediately feel better, seek medical attention”* [88].

The COVID-19 pandemic also added barriers to the public’s access to water for hydration and heat stress prevention. Several articles discussed the implications of reduced potable water access for people experiencing homelessness due to the closure by health authorities of various public facilities due to COVID-19. For example: *“People experiencing homelessness don’t have access to fresh water like you, and I do, and COVID has added an additional barrier to them even accessing public washrooms to find a tap”* [89]. Many public water fountains were also shut down due to COVID-19, with articles citing that the continuation of restricted access was due to requirements for cleaning and staffing shortages. For example, *“The 15 water fountains [in the Capital Regional District] will remain shut off as the humidex is expected to near 40*°*C in the region over the weekend. Phase 2 of B.C.’s [COVID-19] reopening plan requires public fountains to be cleaned no less than once per hour…We are at full capacity for staff work over this weekend; busy with more than full work to do in our many parks which are extremely busy…Each jurisdiction needs to decide what risk they will take and what they can manage”* [90]. The water fountain closures in several locations were reported to have reduced public access to drinking water (a critical and effective heat stress prevention action), disproportionately impacting socially marginalized populations.

The COVID-19 pandemic posed additional challenges to individuals trying to access cool water and engage in heat mitigation behaviors. For example, although indoor and outdoor pools reopened in many cities because of the extreme heat, requirements for *“registering for a swim slot [was] required due to the pandemic”* [91]. In other cities, it was reported that the reopening of wading pools, spray parks, and splash pads was *“delayed by the province’s three-step COVID-19 reopening framework”* [92], which resulted in these facilities not opening until mid-way through the Heat Dome.

### 3.4. COVID-19 Exacerbated the Health Impacts of Cascading Weather Events following the Heat Dome

A series of cascading weather events occurred in British Columbia in the days and weeks following the Heat Dome, including wildfires, poor air quality (i.e., elevated ozone levels, smoke from wildfires), drought, and significant flooding in the heat-affected areas. Some of these cascading weather events (e.g., wildfires) likely resulted from the Heat Dome, whereas others (e.g., atmospheric rivers) did not, but nevertheless impacted the same locations [93]. Responses to address these subsequent weather events were also impacted by the COVID-19 pandemic. For example, in combating the wildfires, a few articles discussed that Canada’s *“sourcing [of] firefighters from some of its usual allies”* [94] was impacted by *“the COVID-19 pandemic…while B.C. has regularly swapped crews with Australia, New Zealand and the United States during times of need, that hasn’t been possible this year. In Australia, they’re on lockdown at the moment [due to COVID-19]”* [94]. Another example is that with the rising temperatures, the ground level ozone also led to air quality alerts in various cities, which subsequently led to articles warning of the combined health concern of poor air quality and extreme heat *“for people with underlying health conditions and respiratory infections, such as COVID-19”* [95].

### 3.5. Heat Impacting COVID-19 Public Health Efforts

Extreme temperatures were also reported to impact the provision and implementation of COVID-19 public health measures. Most articles that touched on this concept communicated the impact of extreme heat on the operation of COVID-19 vaccination and testing clinics. During the Heat Dome, many clinics and testing sites were closed to protect the health and safety of staff and clients from the *“elevated internal temperatures”* [96]. For example, *“the sweltering temperatures throughout much of Western Canada has forced the closure of two COVID-19 vaccination clinics and a testing site in the Vancouver Coastal health region”* [97]. Multiple outdoor pop-up sites were reported to have moved *“indoors to cooler locations in preparation for the extreme heat”* [98], with additional measures being added at other sites, such as *“umbrellas to provide shade for people waiting outside and bottled water and cooling packs…for people who may become overheated”* [99]. A few articles also reported the impact of the extreme heat on the vaccine supply itself, citing the need for health professionals to protect the *“integrity of the temperature-sensitive vaccines”* [100]. Although mentioned infrequently, a few articles recommended the public get vaccinated during the heat wave to bolster the public’s sense of safety when accessing cool public spaces. For example, *“getting vaccinated will make being inside malls or other air-conditioned places much safer for you and for others”* [88].

## 4. Discussion

Health risks were amplified when the COVID-19 pandemic overlapped with EHEs globally [7,11,18]. We believe that this study is the first to use content analysis of digital media articles to examine the intersection of two public health crises, specifically how the co-occurrence of the 2021 Heat Dome and the COVID-19 pandemic in Canada impacted each other. Our novel approach allowed for the interpretation of textual meanings rather than just quantifying textual features (e.g., word frequency) and, importantly, provided insight into how heat health information was disseminated to the public and how the media reported on a health and environmental issue in Canada.

This study found that the news media emphasized the newsworthiness of the intersecting crises by highlighting their compounding burden on health systems in western Canada. During the summer of 2021, the COVID-19 pandemic had already been straining health systems across Canada for over a year, and many media articles conveyed how the 2021 Heat Dome further stretched health services beyond their limits. During the 2021 Heat Dome, the media reported increased emergency call volumes, long wait times for emergency services, and overworked health workers. Previous studies have described how health workers wearing personal protective equipment for COVID-19 experience heat strain [7,11,18,21,31,32,34,35]. However, our study, by analyzing media articles that combined different sources and reported lived-experience stories [43,44,45], showed the mental health effects of EHEs on healthcare providers and workers in other sectors during the COVID-19 pandemic. For instance, health workers were quoted by the media expressing mental fatigue from working in hot environments and attending to increasing calls while feeling criticized for not meeting emergency service demands. Other studies have similarly emphasized that the mental health of nurses and other medical practitioners was impacted as a result of the outbreak [101,102,103]. However, we are unaware of any research that jointly investigated healthcare providers’ mental health during the concurrent extreme heat conditions and pandemic. Although not specifically an occupational study, one recent article by Wilhelmi et al. [38] did find that millions of people in the United States had difficulty mentally coping with or responding to extreme heat because of the direct and indirect effects of the COVID-19 pandemic, and one-third of the population expressed worry about heat when they were at work. Correspondingly, our media analysis highlights the need for better mental health support for the public and workers impacted by concurrent crises [104]. Further, our findings underscore the importance of all levels of government taking proactive actions to consider intersecting health crises in emergency plans and considering investments to support services and businesses during coinciding crises.

The media disseminated conflicting public health recommendations about the 2021 Heat Dome and COVID-19, such as: staying indoors to minimize social contact, choosing outdoor locations if gathering with others to improve ventilation, seeking indoor cool public spaces to escape the heat, and minimizing time spent outdoors to avoid exposure to extreme heat and poor air quality [105]. Although heat mitigation guidance was released in anticipation and during the COVID-19 pandemic [106,107], our findings suggest that the public health experts interviewed for media articles may have been unaware of these resources. The reactive nature of the heat response and the likely impromptu nature of interviews with experts by the media during the 2021 Heat Dome may partly explain the discordance found in public health messaging. However, the factors determining how governments and public health agencies may have prioritized one health crisis above another in their messaging remain unclear. There is also a lack of information on how those who speak about the heat in the media may influence responses by the public. Members of the public interviewed in many articles criticized B.C.’s response as inadequate; the articles expressed concerns regarding the prioritization of COVID-19 in pre-planned speeches from government officials and the lack of community-level preparedness for heat [93]. Although speculative, it is plausible that message fatigue and resistance to persuasion related to COVID-19 may have impacted the dissemination and reception of heat-health messaging during the 2021 Heat Dome, leading to a delayed uptake of heat mitigative actions and/or response by the health system and ultimately the loss of lives [108]. Health authorities must strategize messaging during coinciding crises to emphasize the importance of risk mitigation behaviors while minimizing reluctance—a psychological defense mechanism used in response to a perceived threat or loss of behavioral freedom [109,110]. Further, to ensure consistent public health messaging in the future, federal, provincial, territorial, and jurisdictional health authorities could engage more closely with media producers (e.g., journalists and editors) to help ensure evidence-based interventions are communicated to the public within their local contexts during health crises.

Our results also showed that the media often framed the COVID-19 pandemic as having exacerbated the health impacts of the 2021 Heat Dome. Similar to a recent analysis by Jin and Sanders [40], the news coverage of the 2021 Heat Dome in Canada relayed how COVID-19 restrictions amplified heat health risks through reduced access to cooling strategies. Our results showed building occupancy restrictions, requirements to maintain physical distance, and masking were the main barriers limiting access to or deterring people from cooling centers and other air-conditioned spaces (e.g., malls and libraries). Access to water for bathing and drinking was also reported to have been restricted by COVID-19-driven barriers (e.g., pre-booking swimming and closed public drinking water fountains). These restrictions resulted in additional challenges to protecting people from heat, especially among socially marginalized individuals, as evidenced by reports of reduced potable water access for people experiencing homelessness. Additionally, considering that vulnerabilities to COVID-19 and extreme heat often overlap (e.g., older adults and individuals with co-morbidities) [19], community-level heat health action plans should be adjusted to pre-emptively protect against heat and other concurrent crises to reduce the strain on health systems (e.g., tailored initiatives to support vulnerable groups such as wellness check-ins).

As reported in the articles, many more people in British Columbia and Alberta died from heat than COVID-19 during the 2021 Heat Dome [1,3,4]. Yet, in many articles, the requirements to follow pre-existing pandemic restrictions were prioritized, perhaps confusing the public. As Jin and Sanders [40] note, heat response plans vary greatly between health regions, and clear guidelines for best practices during overlapping EHEs and pandemics remain elusive. A potential solution for ensuring consistent messaging would be to engage all stakeholders involved in heat health messaging at a pan-Canadian level, to pre-emptively develop consistent, evidence-based guidance to help inform the messages they share during future EHEs. Further, when EHEs overlap with other health crises, public health plans optimally should convey that taking the recommended health-protective measure for one public health crisis should not jeopardize one’s health from another crisis.

Given this study included articles published up to seven months after the EHE, we were also able to highlight how the media continued to report (frame) that the COVID-19 pandemic exacerbated the health impacts of weather events that occurred after and due to the 2021 Heat Dome. For instance, due to increased wildfires, poor air quality was observed in British Columbia and Alberta in the months following the EHE. As a result, the media published content communicating that underlying respiratory infections such as COVID-19 can aggravate the health risks associated with exposure to wildfire smoke. Moreover, many articles highlighted how the floods and wildfires that followed the 2021 Heat Dome compounded demand on and stress to the health system. This finding is significant in the context of climate change, as future EHEs and cascading weather events in Canada are likely to again lead the media to communicate during dual crises the strain on health systems and emergency response systems [1]. Consequently, public health officials and other health and public safety system stakeholders should proactively engage with the media to ensure effective content, timing, and prioritization of public health messaging in news coverage during intersecting crises. Additionally, a clear line of communication between health officials and journalists/editors could be established in advance of intersecting crises, which would allow for consistent messaging to be initiated more rapidly and deliberately.

### Limitations

This study has a few limitations. Importantly, we analyzed articles from mass-media outlets, associations, and agency press which contain potential bias from various stakeholders involved in creating news media content [49]. Thus, our findings reflect the interpretations of these sources as content generators. However, as our intent was to broadly explore how the topic is framed/presented to the public, it was not within the scope of this analysis to look specifically at how content differed between media sources or how they approached the inclusion/exclusion of content. Therefore, this poses an opportunity for future work to expand on these findings and explore how different cultural and geographic contexts and political leanings, among other factors, may influence the reporting of intersecting public health crises. The findings are also limited to exploring Canada’s public health communication landscape and do not reflect every North American region affected by the 2021 Heat Dome; the experiences in the United States could have been different and thus warrant investigation in relation to their public health system and communication channels. Another limitation of the current study is the inability to distinguish the voices behind the messaging reported in the captured articles. Given it is not always clear who contributed to the messaging or how they were found (e.g., health officials, academics, journalists, members of the public, and others), future investigations may look to address these gaps by broadening the scope of analysis and seeking additional perspective directly from the knowledge disseminators.

## 5. Conclusions

As part of the most extensive investigation to date to systematically review and content-analyze digital media articles relating to an EHE, this study advances our knowledge of Canada’s public health communication landscape during the intersection of the COVID-19 pandemic and the 2021 Heat Dome. Overall, there was conflicting public health messaging communicated in the news media. Additionally, this study provided insight into the compounding impacts of the EHE and COVID-19 pandemic and how each crisis led to the worsening of health impacts on each other. In the coming decades, health systems and public health management will need to adapt to withstand overlapping threats to public health, including but not limited to EHEs and infectious diseases due to climate change. Findings from this study highlight the need for preparedness plans and developing consistent and evidence-based public health messaging. Health authorities could do this by working with key stakeholders involved in the public and media responses to health crises. Investments could be made to strengthen existing health infrastructure and services to pre-emptively build needed surge capacity, including support for mental health during such events. With the global death tolls from extreme heat and COVID-19 still rising, it is imperative to prioritize and strengthen the communication and design of public health strategies, especially those addressing two or more intersecting health crises, to foster public resilience and readiness.

## Figures and Tables

**Table 1:** Examples of literature investigating extreme heat events and the COVID-19 pandemic.

Country	Articles
Australia	[13,14]
China	[15,16]
France	[17]
Germany	[18,19]
India	[20,21,22]
Iran	[23]
Israel	[24]
Italy	[25]
Japan	[8,15,26,27,28,29,30,31]
Netherlands	[11,32]
Portugal	[15]
Serbia	[33]
Singapore	[21]
United Kingdom	[34,35,36,37]
United States	[38,39,40]

## Data Availability

Data are available from the corresponding author (M.G.) upon reasonable request and signed access agreement.

## Supplementary Materials

The following supporting information can be downloaded at: https://www.mdpi.com/article/10.3390/ijerph20176674/s1, Table S1: Summary of themes and concepts identified in digital media articles discussing the COVID-19 pandemic and the 2021 Heat Dome. Refs. [111,112,113,114,115,116,117,118,119,120,121,122,123,124,125,126] are cited in the Supplementary Materials.

## References

[1] Henderson S.B., McLean K., Lee M., Kosatsky T. (2021). Extreme Heat Events Are Public Health Emergencies. BC Med. J..

[2] British Columbia Coroners Service (2022). Heat-Related Deaths in B.C..

[3] City of Calgary (2022). Summary of Disaster Risk 2021.

[4] B.C. Centre for Disease Control BC COVID-19 Data, 2021. http://www.bccdc.ca/Health-Info-Site/Documents/Guidance-for-Cooling-Centres-COVID-19.pdf.

[5] Government of Alberta COVID-19 Alberta Statistics: Interactive Aggregate Data on COVID-19 Cases in Alberta, 2022. https://www.alberta.ca/stats/covid-19-alberta-statistics.htm#data-export.

[6] Government of Canada COVID-19: Canada’s Response, 2020. https://www.canada.ca/en/public-health/services/diseases/2019-novel-coronavirus-infection/canadas-reponse.html.

[7] Daanen H., Bose-O’Reilly S., Brearley M., Flouris D.A., Gerrett N.M., Huynen M., Jones H.M., Lee J.K.W., Morris N., Norton I. (2021). COVID-19 and Thermoregulation-Related Problems: Practical Recommendations. Temperature.

[8] Otani S., Ishizu S.F., Masumoto T., Amano H., Kurozawa Y. (2021). Elevated Heat Stroke Risk in Older Adults Indirectly Caused by COVID-19 Restrictions in a Provincial Prefecture of Japan. Med. Sci. Forum.

[9] Watts N., Amann M., Arnell N., Ayeb-Karlsson S., Beagley J., Belesova K., Boykoff M., Byass P., Cai W., Campbell-Lendrum D. (2021). The 2020 Report of The Lancet Countdown on Health and Climate Change: Responding to Converging Crises. Lancet.

[10] Bedford J., Farrar J., Ihekweazu C., Kang G., Koopmans M., Nkengasong J. (2019). A New Twenty-First Century Science for Effective Epidemic Response. Nature.

[11] Bongers C.C.W.G., de Korte J.Q., Zwartkruis M., Levels K., Kingma B.R.M., Eijsvogels T.M.H. (2022). Heat Strain and Use of Heat Mitigation Strategies among COVID-19 Healthcare Workers Wearing Personal Protective Equipment—A Retrospective Study. Int. J. Environ. Res. Public Health.

[12] Sousa P.M., Trigo R.M., Russo A., Geirinhas J.L., Rodrigues A., Silva S., Torres A. (2022). Heat-Related Mortality Amplified during the COVID-19 Pandemic. Int. J. Biometeorol..

[13] Hospers L., Smallcombe J.W., Morris N.B., Capon A., Jay O. (2020). Electric Fans: A Potential Stay-at-Home Cooling Strategy during the COVID-19 Pandemic This Summer?. Sci. Total Environ..

[14] Stephens D., Brearley M., Vermeulen L. (2022). Heat Health Management in a Quarantine and Isolation Facility in the Tropics. Prehospital Disaster Med..

[15] Uryu S., Tanoue Y., Nomura S., Matsuura K., Makiyama K., Kawashima T., Yoneoka D., Eguchi A., Kawamura Y., Gilmour S. (2021). Trends in Emergency Transportation Due to Heat Illness under the New Normal Lifestyle in the COVID-19 Era, in Japan and 47 Prefectures. Sci. Total Environ..

[16] Yuan N., Yang W.-X., Lu J.-L., Lv Z.-H. (2021). Investigation of Adverse Reactions in Healthcare Personnel Working in Level 3 Barrier Protection PPE to Treat COVID-19. Postgrad. Med. J..

[17] Pascal M., Lagarrigue R., Laaidi K., Boulanger G., Denys S. (2021). Have Health Inequities, the COVID-19 Pandemic and Climate Change Led to the Deadliest Heatwave in France since 2003?. Public Health.

[18] Jegodka Y., Lagally L., Mertes H., Deering K., Schoierer J., Buchberger B., Bose-O’Reilly S. (2021). Hot Days and Covid-19: Online Survey of Nurses and Nursing Assistants to Assess Occupational Heat Stress in Germany during Summer 2020. J. Clim. Chang. Health.

[19] Bose-O’Reilly S., Daanen H., Deering K., Gerrett N., Huynen M.M.T.E., Lee J., Karrasch S., Matthies-Wiesler F., Mertes H., Schoierer J. (2021). COVID-19 and Heat Waves: New Challenges for Healthcare Systems. Environ. Res..

[20] Purushothaman P.K., Priyangha E., Vaidhyswaran R. (2021). Effects of Prolonged Use of Facemask on Healthcare Workers in Tertiary Care Hospital During COVID-19 Pandemic. Indian J. Otolaryngol. Head Neck Surg..

[21] Lee J., Venugopal V., Latha P.K., Alhadad S.B., Leow C.H.W., Goh N.Y.D., Tan E., Kjellstrom T., Morabito M., Lee J.K.W. (2020). Heat Stress and Thermal Perception amongst Healthcare Workers during the COVID-19 Pandemic in India and Singapore. Int. J. Environ. Res. Public Health.

[22] Golechha M., Panigrahy R.K. (2020). COVID-19 and Heatwaves: A Double Whammy for Indian Cities. Lancet Planet. Health.

[23] Pourkarim M.R., Thijssen M., Lemey P., Vandamme A.-M., Van Ranst M. (2020). Air Conditioning System Usage and SARS-CoV-2 Transmission Dynamics in Iran. Med. Hypotheses..

[24] Finzi Y., Ganz N., Limon Y., Langer S. (2021). The next Big Earthquake May Inflict a Multi-Hazard Crisis—Insights from COVID-19, Extreme Weather and Resilience in Peripheral Cities of Israel. Int. J. Disaster Risk Reduct..

[25] Morabito M., Messeri A., Crisci A., Pratali L., Bonafede M., Marinaccio A. (2020). Heat Warning and Public and Workers’ Health at the Time of COVID-19 Pandemic. Sci. Total Environ..

[26] Kanda J., Miyake Y., Umehara T., Yoshiike S., Fujita M., Hayashida K., Hifumi T., Kaneko H., Kobayashi T., Kondo Y. (2022). Influence of the Coronavirus Disease 2019 ( COVID-19) Pandemic on the Incidence of Heat Stroke and Heat Exhaustion in Japan: A Nationwide Observational Study Based on the Heatstroke STUDY 2019 (without COVID-19) and 2020 (with COVID-19). Acute Med. Surg..

[27] Shimizu K., Gilmour S., Mase H., Le P.M., Teshima A., Sakamoto H., Nomura S. (2021). COVID-19 and Heat Illness in Tokyo, Japan: Implications for the Summer Olympic and Paralympic Games in 2021. Int. J. Environ. Res. Public Health.

[28] Seposo X., Madaniyazi L., Ng C.F.S., Hashizume M., Honda Y. (2021). COVID-19 Pandemic Modifies Temperature and Heat-Related Illness Ambulance Transport Association in Japan: A Nationwide Observational Study. Environ. Health.

[29] Nakahara S., Kanda J., Miyake Y., Sakamoto T. (2021). High Incidence of Heat Illness and the Potential Burden on the Health Care System during the COVID-19 Pandemic. Lancet Reg. Health—West. Pac..

[30] Hatakeyama K., Ota J., Takahashi Y., Kawamitsu S., Seposo X. (2021). Effect of the COVID-19 Pandemic on Heatstroke-Related Ambulance Dispatch in the 47 Prefectures of Japan. Sci. Total Environ..

[31] Unoki T., Tamoto M., Ouchi A., Sakuramoto H., Nakayama A., Katayama Y., Miyazaki S., Yamada T., Fujitani S., Nishida O. (2020). Personal Protective Equipment Use by Health-care Workers in Intensive Care Units during the COVID-19 Pandemic in Japan: Comparative Analysis with the PPE-SAFE Survey. Acute Med. Surg..

[32] de Korte J.Q., Bongers C.C.W.G., Catoire M., Kingma B.R.M., Eijsvogels T.M.H. (2022). Cooling Vests Alleviate Perceptual Heat Strain Perceived by COVID-19 Nurses. Temperature.

[33] Milošević D., Middel A., Savić S., Dunjić J., Lau K., Stojsavljević R. (2022). Mask Wearing Behavior in Hot Urban Spaces of Novi Sad during the COVID-19 Pandemic. Sci. Total Environ..

[34] Davey S.L., Lee B.J., Robbins T., Randeva H., Thake C.D. (2021). Heat Stress and PPE during COVID-19: Impact on Healthcare Workers’ Performance, Safety and Well-Being in NHS Settings. J. Hosp. Infect..

[35] Foster J., Hodder S.G., Goodwin J., Havenith G. (2020). Occupational Heat Stress and Practical Cooling Solutions for Healthcare and Industry Workers During the COVID-19 Pandemic. Ann. Work Expo. Health.

[36] Mahase E. (2022). Covid-19: High Prevalence and Lack of Hospital Beds Putting “Intense Pressure” on Ambulances. BMJ.

[37] Lo Y.T.E., Mitchell D.M., Thompson R., O’Connell E., Gasparrini A. (2022). Estimating Heat-Related Mortality in near Real Time for National Heatwave Plans. Environ. Res. Lett..

[38] Wilhelmi O.V., Howe P.D., Hayden M.H., O’Lenick C.R. (2021). Compounding Hazards and Intersecting Vulnerabilities: Experiences and Responses to Extreme Heat during COVID-19. Environ. Res. Lett..

[39] Bock J., Srivastava P., Jessel S., Klopp J.M., Parks R.M. (2021). Compounding Risks Caused by Heat Exposure and COVID-19 in New York City: A Review of Policies, Tools, and Pilot Survey Results. J. Extreme Events.

[40] Jin A.S., Sanders K.T. (2022). Analyzing Changes to U.S. Municipal Heat Response Plans during the COVID-19 Pandemic. Environ. Sci. Policy.

[41] Caulfield T., Clark M.I., McCormack J.P., Rachul C., Field C.J. (2014). Representations of the Health Value of Vitamin D Supplementation in Newspapers: Media Content Analysis. BMJ Open.

[42] Kim S.-H., Scheufele D.A., Shanahan J. (2002). Think about It This Way: Attribute Agenda-Setting Function of the Press and the Public’s Evaluation of a Local Issue. Journal. Mass Commun. Q..

[43] Hopke J.E. (2020). Connecting Extreme Heat Events to Climate Change: Media Coverage of Heat Waves and Wildfires. Environ. Commun..

[44] Lowrey W., Evans W., Gower K.K., Robinson J.A., Ginter P.M., McCormick L.C., Abdolrasulnia M. (2007). Effective Media Communication of Disasters: Pressing Problems and Recommendations. BMC Public Health.

[45] Weathers M.R., Kendall B.E. (2016). Developments in the Framing of Climate Change as a Public Health Issue in US Newspapers. Environ. Commun..

[46] Sheridan S.C. (2007). A Survey of Public Perception and Response to Heat Warnings across Four North American Cities: An Evaluation of Municipal Effectiveness. Int. J. Biometeorol..

[47] Strauss N., Painter J., Ettinger J., Doutreix M.-N., Wonneberger A., Walton P. (2022). Reporting on the 2019 European Heatwaves and Climate Change: Journalists’ Attitudes, Motivations and Role Perceptions. J. Pract..

[48] Tetzlaff E., Goulet N., Gorman M., Richardson G., Enright P., Meade R., Kenny G. (2023). Hot Topic: A Systematic Review and Content Analysis of Heat-Related Messages during the 2021 Heat Dome in Canada. J. Public Health Manag. Pract..

[49] Bohr J. (2020). Reporting on Climate Change: A Computational Analysis of U.S. Newspapers and Sources of Bias, 1997–2017. Glob. Environ. Chang..

[50] Philip S., Kew S., van Oldenborgh G. (2023). Rapid Attribution Analysis of the Extraordinary Heat Wave on the Pacific Coast of the US and Canada June 2021. Eur. Syst. Dyn..

[51] Fleiss J., Levin B., Paik M. (2003). Statistical Methods for Rates & Proportions.

[52] McIntyre G. (2021). With Heat Warnings in Effect, B.C. Businesses Take Measures to Protect Employees and Customers.

[53] The Chilliwack Progress (2021). A Year of News Like No Other.

[54] Scoffield H. (2021). Lets Hope We Learned the Lessons of COVID-19 Because the Next Crisis Has Arrived.

[55] Herring J. (2021). Dangerous and Historic’ Heat Wave Set to Hit City.

[56] The Weather Network (2021). ‘Off the Charts’ Heat Wave Continues to Stifle Western Canada.

[57] Penticton Western News (2021). A Look Back on the Fire Fight of Summer 2021.

[58] Griffin K. (2021). B.C.’s Extreme Heat Wave Emergencies Linked to Health of Ecosystem.

[59] Waters A. (2021). COVID Spread Delays Surgeries in Kelowna.

[60] Harnett C.E. (2021). Island Residents Asked to Avoid Emergency Rooms If Possible as Hospitals See Spike in Visits.

[61] Chan C. (2021). Heat Wave Linked to Massive Spike in Sudden Deaths across Lower Mainland.

[62] BC Wildfire Service (2021). BC Wildfires Update for July 10.

[63] Rebot K. (2022). Third NewsWatch.

[64] Woo A. (2022). Overdose Calls in British Columbia Rise 31% in 2021.

[65] Henderson J. (2021). Dry and Fried’ Crops across Province Wither under Heat.

[66] Norwell J. (2021). This Kamloops Restaurateur is Closing His Business for a Week to Give His Staff a Mentalhealth Break.

[67] Whitehouse S. (2021). August Opens with Singleday Heat Records.

[68] Mochrie P. (2021). Email: Extreme Heat Alert.

[69] Fraser Health Authority (2021). Extreme Heat Alert.

[70] The Vancouver Sun (2021). B.C. Heat Wave Update for June 28: Lytton Smashes Canadian Heat Record Again with 47.5 C|Record Consumption for BC Hydro|Public Schools to Stay Closed Monday.

[71] Kopecky A. (2021). The Record-Setting Day When Global Heating Surpassed COVID-19 as the Existential Crisis.

[72] Wines A. (2021). As the Heat Dome Takes Lives Canadian Banks Must Acknowledge Their Role in Climate Change.

[73] Elliott L. (2021). Coronavirus What Happened in Canada and around the World on Saturday.

[74] First Nations Health Authority (2021). Important Health Advice During BC’s Heat Advisory.

[75] DeMeer A. (2021). Record Breaking Temperatures Trigger Safety Alerts.

[76] Vancouver Coastal Health (2021). A Heat Warning Has Been Issued for Aug. 12 to Aug. 15.

[77] Vancouver Sun (2021). B.C.’s Inadequate Response to Heat Wave Heightened Risks for Vulnerable People: Report.

[78] Palmer V. (2021). How NDP Gov’t Handled Heat Wave and Wildfires Needs to be Looked Into.

[79] Kay M. (2021). BC NDP Slow to Respond to Crisis.

[80] Ballard J. (2021). She Died Alone in Her Apartment during BCs Heat Wave Her Family Says Her Death Was Preventable.

[81] Hasegawa R., Kotyk A. (2021). Heat Leads to Closure of All Public Schools, Some Post-Secondary in B.C.’s Lower Mainland.

[82] Sciarpelletti L. (2021). Heat Warning in Effect for Regina Dozens of Cooling Spaces Available.

[83] Leisure Services (2021). Cooling Centre at Sportsplex Main Arena.

[84] Johnson L. (2021). Albertans Asked to Conserve Energy as Province Breaks Record Second Day in a Row Amid Heat Wave.

[85] Joannou A. (2021). Agencies Gear up for Heat.

[86] Herring J. (2021). City Set to Sizzle.

[87] First Nations Health Authority (2021). Enjoy the Hot Weather this Summer, though Think Ahead to Stay Healthy and Safe!.

[88] Babych S. (2021). Scorched; Calgary Works to Protect Vulnerable from Record-Breaking Heat Wave.

[89] Green K. (2021). Water Fountains Refill Stations to Remain Shut off in CRD Parks Despite Heatwave.

[90] Chan C. (2021). BC Heatwave: Here’s 15 Ways to Beat the Heat and Stay Cool.

[91] Brown D. (2021). Hot Sticky Weather to Linger until Midweek.

[92] Environment and Climate Change Canada (2022). Canada’s Top 10 Weather Stories of 2021.

[93] Johansen N. (2021). Firefighters from Australia New Zealand US Unable to Help BC Battle Wildfires.

[94] Canadian Press (2021). Air Quality Advisory in Effect for Parts of Vancouver, Fraser Valley Amid Heatwave Vernon Matters.

[95] Dayal P. (2021). Scorching Heat Imperils Elderly, Vulnerable; Health Authorities Urge High-Risk Groups, Outdoor Workers to Stay Hydrated as Temperatures Soar.

[96] Reis N. (2021). Ninth NewsWatch: WEA, Heat, Nights.

[97] The Star (2021). Todays Coronavirus News Massive Vaccine Drive at Scotiabank Arena in Toronto Aims to Shatter North America Record.

[98] Galligan R. (2021). All VCH Vaccination Clinics Now Welcome Drop-in First Dose Appointments.

[99] Jang B., Woo A. (2021). Health Warnings Issued Amid Heat Wave.

[100] Sampaio F., Sequeira C., Teixeira L. (2020). Nurses’ Mental Health During the Covid-19 Outbreak: A Cross-Sectional Study. J. Occup. Environ. Med..

[101] Liu Z., Han B., Jiang R. (2020). Mental Health Status of Doctors and Nurses during COVID-19 Epidemic in China. https://ssrn.com/abstract=3551329.

[102] Lake E.T., Narva A.M., Holland S., Smith J.G., Cramer E., Rosenbaum K.E.F., French R., Clark R.R.S., Rogowski J.A. (2022). Hospital nurses’ moral distress and mental health during COVID-19. J. Adv. Nurs..

[103] Bratu A., Card K.G., Closson K., Aran N., Marshall C., Clayton S., Gislason M.K., Samji H., Martin G., Lem M. (2022). The 2021 Western North American heat dome increased climate change anxiety among British Columbians: Results from a natural experiment. J. Clim. Change Health.

[104] Public Health Agency of Canada COVID-19: Guidance on Indoor Ventilation during the Pandemic. Government of Canada, 2023. https://www.canada.ca/en/public-health/services/diseases/2019-novel-coronavirus-infection/guidance-documents/guide-indoor-ventilation-covid-19-pandemic.html.

[105] Global Heat Health Information Network Protecting Health from Hot Weather during the COVID-19 Pandemic, 2020. https://ghhin.org/wp-content/uploads/technical-brief-COVID-and-Heat-finalv2-1.pdf.

[106] Health Canada Extreme Heat and COVID-19, 2022. https://www.canada.ca/en/health-canada/services/sun-safety/extreme-heat-covid-19.html.

[107] Islam A.K.M.N., Laato S., Talukder S., Sutinen E. (2020). Misinformation sharing and social media fatigue during COVID-19: An affordance and cognitive load perspective. Technol. Forecast. Soc. Chang..

[108] Guan M., Li Y., Scoles J.D., Zhu Y. (2022). COVID-19 Message Fatigue: How Does It Predict Preventive Behavioral Intentions and What Types of Information are People Tired of Hearing About?. Health Commun..

[109] Reynolds-Tylus T. (2019). Psychological Reactance and Persuasive Health Communication: A Review of the Literature. Front. Commun..

[110] Woo A., Freeze C. (2021). Health Care System Hit Hard by Staffing Shortages Amid COVID-19 Surge.

[111] CTV News (2021). Hope BC under a State of Emergency.

[112] Dickson C. (2021). British Columbians Head to Parks Crank up the AC during Recordbreaking Heat Wave.

[113] Mason G. (2021). BC Has Managed Its Twin Crises Abysmally.

[114] Saddle Hills County (2021). Heat Wave Safety Tips.

[115] Phillips B. (2021). Vernon to Open Kal Tire Place as Cooling Centre during Heatwave.

[116] City of Cranbrook (2021). Residents Reminded to Bring Their Own Water to City Parks and Facilities During Heat Wave.

[117] Kloster D. (2021). Hottest Temperature on Record Hits Capital Region.

[118] Haluschak E., Farrell T. (2021). All-Time Heat Record Set in Comox, Daily Records Smashed All Weekend.

[119] Kilian C. (2021). Seven Big Warnings from the Killer Heat Wave.

[120] Haliburton, Kawartha, Pine Ridge District Health Unit (2021). Hot Weather—Beat the Extreme Heat.

[121] Galloway M. (2021). Designing Homes Equipped to Beat the Heat.

[122] WorkSafe BC (2021). WorkSafe Tools for Building Safer Workplaces.

[123] British Columbia Assembly of First Nations (2021). Climate Change & Water Newsletter.

[124] Crawford T. (2021). BC Climate News Dec 27–Jan 2 2022: Premier John Horgan Says 2021 Will Be Remembered as ‘Year That Climate Change Arrived on Our Doorsteps’|Climate- Related Disasters Causing Surge in Mental Health Problems|Trudeau Cites Climate Change a Key Take-Away from 2021.

[125] The Chilliwack Progress (2021). Memorable Images of 2021.

[126] Farrell T. (2021). Comox Valley Vaccination Bookings Being Rescheduled Due to Heat Wave.

