# Supplementary material for: The Intersection of the COVID-19 Pandemic and the 2021 Heat Dome in Canadian Digital News Media: A Content Analysis
Source: Int J Environ Res Public Health. 2023 Aug 29;20(17):6674. doi: 10.3390/ijerph20176674 (PMC10488163; doi:10.3390/ijerph20176674)
Supplement: Supplementary file 1 [file ijerph-20-06674-s001.zip › ijerph-2545050-supplementary.pdf]

Supplemental Material

The Intersection of the COVID-19 Pandemic and the 2021 Heat Dome in Canadian Digital News Media: A Content Analysis

Table 1. Summary of themes and concepts identified in digital media articles discussing the COVID-19 pandemic and the 2021 Heat Dome.

Theme	Concepts	Positive Indicators (keywords) of Concept	Textual Example
**Communicating the Burden of Multiple Intersecting Public Health Crises**	Burden on Health Systems (n=55)	ambulances, deaths, dispatch centre, dispatchers, doctors, emergency care, emergency departments, emergency response system, emergency rooms, emergency services, excess mortality, first responders, health authority, healthcare crisis, health care services, healthcare system, health conditions, health professionals, hospitals, long-term care, nurses, nursing homes, paramedics, pharmaceutical supply, physicians, provincial health officers, public health crises, public health emergencies	*"B.C.'s emergency response system had faced staffing shortages and high call volumes long before Omicron. At the height of last summer's heat wave, some callers to 911 reported busy signals and waits of more than 10 hours for ambulances to arrive."* [110]
	Burden on Mental Health (n=47)	adversity, afraid, anguish, anxiety, burn out, challenging times, cognitive well-being, concerns, difficult times, faith, fatigue, fear, feeling helpless, grief, heartache, hope, hysteria, mental health, mentally taxing, overwhelmed, perseverance, resilience, stressed, traumatic, triggered, unrest, worry	*"The anxiety's very high. I mean, it's been building over the year, you know, with COVID-19, the heat dome, the forest fires and now this has just compounded and added to everybody's anxiety."* [111]
**Prioritizing Crises & Conflicting Public Health Messaging**	Prioritizing Extreme Heat over COVID-19 (n=18)	back seat, bigger, exceed, greater, outweigh, overshadowed, precedence over, surpassed	*"Health authorities across the province say that the risks associated with extreme heat outweigh those associated with COVID-19, at this time."* [112]
	Failure in Responding to Extreme Heat (n=11)	afterthought, caught flatfooted, caught up, failed to adequately warn, less concerned, preoccupied, slow to respond, too busy, weary government	*"Premier John Horgan's response to it all was not good. He admitted to being caught flatfooted by the intensity of the heat wave, happening as it did while the province was preparing for its pandemic reopening. He offended many when he suggested deaths from heat were inevitable."* [113]
	Conflicting Health Messaging (n=4)	n/a	*"We would prefer that people avoid the exposure to extreme heat outdoors and we would prefer people stay hydrated and cool and eat the right foods, said Hasell. We also realize that after a year-and-a-half of COVID restrictions, if people can take advantage of the weather and gather, they will do it. But it is super important to pay attention when it comes to smoke or heat."* [67]
**COVID-19 Exacerbated the Health Impacts of Extreme Heat**	Physical & Social Distancing (n=80)	capacity limits, capacity restrictions, COVID-19 capacity rules, COVID-related occupancy restrictions, crowding, gathering outside, limited capacity, limited contact, limited seating, physical distancing, physically apart, occupancy limits, reduce face-to-face interactions, social distancing, stay apart, two metres distance	*"Take frequent breaks from heat, spending time indoors. If you are going to be indoors in a public building, such as a mall, be sure to respect and follow all COVID requirements, including physical distancing and considering masks."* [114]

	Masking (n=71)	face covering, mask	*"The Interior, Fraser and Vancouver Coastal health authorities say that anyone having trouble breathing while wearing a mask, inside or outside, should remove it immediately." [112]*
	Generic COVID-19 Restrictions (n=57)	COVID-19 precautions, COVID protocol, COVID-19 restrictions, COVID safety protocols, health protocols, pandemic reopening plan, pandemic restrictions, public health guidelines, public health restrictions, shackles	*"She added officials are balancing COVID restrictions with the need for people to stay cool." [55]*
	COVID-19 Measures at Cooling Centres (n=56)	capacity limits, capacity restrictions, contact tracing, COVID precautions, COVID-19 capacity rules, COVID-19 restrictions, COVID-19 safety protocols, filtration, hand washing, health restrictions, limited seating, masks, misting fans, physical distancing, plexiglass barriers, public health guidelines, social distancing	*"In accordance with COVID-19 safety protocols, cooling centre attendees will be required to provide their name and contact information for contact tracing, wear masks and continue to practice physical distancing while inside the facility."* [115]
	Water Access (n=29)	bottled water, drinking fountain, drinking water, fresh water, hydrated, pools, public fountain, splash pads, splash parks, spray pads, spray parks, swimming spot, tap, water, water fountain, water station, waterplay options	*"Due to the COVID-19 pandemic, the City closed its various water fountains and bottle fill stations around the community. Given the current pandemic restrictions, those water stations remain closed. Residents should be well prepared by bringing their own water to ensure they remain hydrated during the heat wave."* [116]
	Heat Mitigation Efforts (n=27)	air conditioning, air-conditioned spaces, bottled water, cooler indoor venue, cooler locations, cooling packs, fans, hat, misting fan, portable air conditioners, shade, sites with cooler temperatures, sunscreen, tenting, umbrellas	*"Island Health brought in portable air conditioners, extra fans and tenting at entrances in attempts to cool some arena venues."* [117]
	Ventilation & Fan Use (n=17)	air flow, blowing air, circulation of fresh air, fans, filtration, recirculation of indoor air, ventilation, windows	*"Schreiner noted they also assisted with the vaccine clinic at Glacier Gardens Arena throughout the weekend to help cool down the space with a misting fan, however they had to ensure they were not blowing air too far due to COVID-19."* [118]
	Travel Restrictions (n=10)	COVID-19 travel restrictions, shackles, travel bans, travelling restrictions, unable to travel due to the pandemic	*"The relatively normal temperatures of early July, together with the relaxed COVID-19 travel restrictions, might encourage us to forget the last week of June and enjoy the rest of the summer. But the heat wave wasn't a one-off."* [119]
	Symptom Screening (n=10)	COVID-19 safety checks, COVID-19 screening, COVID-19 symptoms, rapid COVID-19 testing, required to quarantine, tested for the coronavirus	*"If you experience any heat-related illness or symptoms, seek immediate medical attention. But remember, if you have symptoms of COVID-19, please call ahead if possible to inform health care providers or first responders so they can take appropriate preventive measures."* [120]

	Sanitization & Cleaning (n=8)	clean, disinfect, frequently washing your hands, hand hygiene, precautions to sanitize, respiratory etiquette, sanitizing, sanitizing stations	*"Water fountains and refill stations throughout Capital Regional District parks and trails will remain shut off during this weekend's expected heat wave, given a lack of resources to clean them to COVID pandemic standards."* [89]
	Work from Home (n=5)	home-based workers, returning to "traditional work styles", working from home, working remotely	*"We live in a townhouse and it's pretty hot in there and because of the pandemic, we're working from home. And so I called in to work this morning and said we can work from home and I'm not going to have our son in school either. So we had to take today off and just looking for shade."* [121]
**COVID-19 Exacerbated the Health Impacts of Cascading Weather Events following the Heat Dome**	Wildfires (n=84)	fires, forest fires, megafires, wildfires	*"British Columbia is having a tough time sourcing firefighters from some of its usual allies thanks to the COVID-19 pandemic and widespread wildfires elsewhere in the world."* [122]
	Flooding (n=46)	atmospheric rivers, flooding, floods, torrential rains	*"In addition to the ongoing pandemic, there were weather events that affected our businesses and our families, such as the heat dome and Lytton fire, and the atmospheric river and highway collapse."* [123]
	Drought (n=11)	drought	*"The impacts of global warming combined with local conditions have brought heatwaves, wildfires, drought conditions, and floods in the early summer. These events, in addition to the ongoing COVID-19 pandemic… have further strained communities across BC."* [124]
	Poor Air Quality (n=8)	air pollution, air quality issues, heavy smoke, poor air quality, wildfire smoke	*"The start of the pandemic was really hard on people but when I really noticed the spike in my own practice was when the wildfire smoke would come, said Lem, who is an assistant professor in UBC's faculty of medicine."* [125]
	Extreme Cold (n=4)	bitterly cold winter temperatures, crazy snow, deep freeze, early freeze	*"B.C. was hit hard this year with atmospheric rivers, a heat dome, and bitterly cold winter temperatures – all during an ongoing pandemic."* [126]
**Heat Impacting COVID-19 Public Health Efforts**	Vaccination & Testing Postponements (n=98)	COVID-19 safety checks, immunization appointment, immunization centre, immunization clinic, immunization rollout, mass immunization, pop-up clinic, testing clinic, testing services, vaccination centre, vaccination clinic, vaccination schedule, vaccination site, vaccine appointment, vaccine clinic	*"Island Health has decided to rebook all afternoon appointments at Glacier Gardens in Comox scheduled for Wednesday, June 30, due to the extreme heat wave affecting British Columbia. The Glacier Gardens vaccination centre was closed all day Tuesday, June 29, for the same reason. The facility has to close early Monday, due to heat concerns from staff inside the building."* [127]
	Vaccine Storage (n=4)	integrity of the temperature-sensitive vaccines, vaccine supply	*"Despite mitigation measures, the high temperatures and lack of air conditioning are impacting the safety of patients, staff and our vaccine supply if these sites remain open."* [99]

	Vaccine Protection (n=2)	vaccination; immunization	*"Getting vaccinated will make being inside malls or other air-conditioned places much safer for you and for others."* [87]

Notes: Positive indicators for textual coding represent the actual elements a text was composed of that directed its inclusion in a specific component category. The numbers in square brackets provided with the quotations represent the reference number for the cited article and are listed below.
